# Supplementary material for: Anogenital HPV-Related Cancers in Women: Investigating Trends and Sociodemographic Risk Factors
Source: Cancers (Basel). 2024 Jun 8;16(12):2177. doi: 10.3390/cancers16122177 (PMC11202297; doi:10.3390/cancers16122177)
Supplement: Supplementary file 1 [file cancers-16-02177-s001.zip › cancers-3053372-supplementary.pdf]

**Table 1.** Table summarising the demographics of patients with anogenital cancer between 2014 and 2020.

| Cancers | Anal | Cervical | Vaginal | Vulval | Total |
|---|---|---|---|---|---|
| **N** | 5852 | 17854 | 1433 | 7754 | 32893 |
| **% of Total** | 17.8% | 54.3% | 4.4% | 23.6% | 100% |
| **Age Groups** | | | | | |
| < 40y | 145 (2.5%) | 7055 (39.5%) | 48 (3.3%) | 211 (92.7%) | 7459 (22.7%) |
| 40 – 54y | 1196 (20.4%) | 5110 (28.6%) | 231 (16.1%) | 1170 (15.1%) | 7707 (23.4%) |
| 55 – 74y | 3043 (52.0%) | 3901 (21.8%) | 633 (44.2%) | 3029 (39.1%) | 10606 (32.2%) |
| 75+y | 1468 (25.1%) | 1788 (10.0%) | 521 (36.4%) | 3344 (43.1%) | 7121 (21.6%) |
| **Ethnicity** | | | | | |
| Asian | 38 (0.1%) | 447 (2.5%) | 46 (3.2%) | 143 (1.8%) | 674 (2.0%) |
| Black | 73 (1.2%) | 286 (1.6%) | 46 (3.2%) | 80 (1.0%) | 485 (1.5%) |
| Mixed and Other | 75 (1.3%) | 510 (2.9%) | 18 (1.3%) | 94 (1.2%) | 697 (2.1%) |
| Unknown | 209 (3.6%) | 1108 (6.2%) | 49 (3.4%) | 317 (4.1%) | 1683 (5.1%) |
| White | 5457 (93.3%) | 15503 (86.8%) | 1274 (88.9%) | 7120 (91.8%) | 29354 (89.2%) |
| **Deprivation** | | | | | |
| 1 | 1246 (21.3%) | 4694 (26.3%) | 334 (23.3%) | 1678 (21.6%) | 7952 (24.2%) |
| 2 | 1223 (20.9%) | 3883 (21.7%) | 279 (19.5%) | 1556 (20.1%) | 6941 (21.1%) |
| 3 | 1188 (20.3%) | 3476 (19.5%) | 301 (21.0%) | 1585 (20.4%) | 6550 (19.9%) |
| 4 | 1116 (19.1%) | 3138 (17.6%) | 270 (18.8%) | 1545 (19.9%) | 6069 (18.5%) |
| 5 | 1079 (18.4%) | 2663 (14.9%) | 249 (17.4%) | 1390 (17.9%) | 5381 (16.4%) |
| **Stage** | | | | | |
| 1 | 674 (14.4%) | 5592 (51.9%) | 223 (23.9%) | 3753 (65.2%) | 10242 (46.2%) |
| 2 | 1331 (28.4%) | 2428 (22.5%) | 199 (21.3%) | 440 (7.6%) | 4398 (19.9%) |
| 3 | 2161 (46.2%) | 1169 (10.9%) | 218 (23.4%) | 1104 (19.2%) | 4652 (21.0%) |
| 4 | 515 (11.0%) | 1584 (14.7%) | 293 (31.4%) | 462 (8.0%) | 2854 (12.9%) |
| **Years** | | | | | |
| 2014 | 735 (12.6%) | 2529 (14.2%) | 204 (14.2%) | 1069 (13.8%) | 4537 (13.8%) |
| 2015 | 870 (14.9%) | 2466 (13.8%) | 197 (13.7%) | 1098 (14.2%) | 4631 (14.1%) |
| 2016 | 855 (14.6%) | 2568 (14.4%) | 208 (14.5%) | 1071 (13.8%) | 4702 (14.3%) |
| 2017 | 825 (14.1%) | 2564 (14.4%) | 205 (14.3%) | 1110 (14.3%) | 4704 (14.3%) |
| 2018 | 849 (14.5%) | 2660 (14.9%) | 196 (13.7%) | 1195 (15.4%) | 4900 (14.9%) |
| 2019 | 878 (15.0%) | 2707 (15.2%) | 218 (15.2%) | 1169 (15.1%) | 4972 (15.1%) |
| 2020 | 840 (14.4%) | 2360 (13.2%) | 205 (14.3%) | 1042 (13.4%) | 4447 (13.5%) |

**Table 2.** Table summarising average age-specific, ethnicity—specific, deprivation—specific and staging—specific incidences as well as average annual percentage changes (AAPC) in incidence between 2014—2020 for all anogenital cancers. Note that ethnicity—specific incidences are not available for the year 2020. The median and lower and upper quartile values are reported for the average incidences, incidences are per 100,000 people. The mean % change and standard deviation are reported for the average yearly percentage change.

| | **Anal Cancer** | | **Cervical Cancer** | | **Vaginal Cancer** | | **Vulval Cancer** | |
|---|---|---|---|---|---|---|---|---|
| | **Incidence** | **% Change** | **Incidence** | **% Change** | **Incidence** | **% Change** | **Incidence** | **% Change** |
| **General** | 4.2 (4.1, 4.3) | 1.7 ± 8.3 | 12.9 (12.7, 13.1) | -1.7 ± 6.15 | 1.0 (1.0, 1.1) | -0.5 ± 6.6 | 5.6 (5.4, 5.7) | -1.0 ± 6.3 |
| **Age Specific** | | | | | | | | |
| <40y | 0.4 (0.3, 0.4) | 12.7 ± 53.2 | 18.3 (18.0, 18.7) | -3.4 ± 6.0 | 0.1 (0.1, 0.2) | 25.4 ± 89.3 | 0.5 (0.4, 0.6) | 18.2 ± 39.9 |
| 40-54 y | 3.0 (2.9, 3.2) | 2.6 ± 11.8 | 12.9 (12.3, 13.4) | 0.6 ± 8.6 | 0.6 (0.5, 0.6) | 2.3 ± 13.7 | 2.9 (2.8, 3.1) | -1.0 ± 8.6 |
| 55-74y | 7.3 (6.9, 7.5) | 3.1 ± 11.9 | 9.5 (8.9, 9.6) | 2.5 ± 5.5 | 1.5 (1.5, 1.6) | 2.4 ± 11.7 | 7.1 (7.0, 7.3) | -0.2 ± 7.4 |
| 75+y | 8.0 (7.7, 8.1) | 1.6 ± 15.5 | 9.6 (9.5, 9.7) | -5.4 ± 7.9 | 2.8 (2.8, 2.9) | -2.2 ± 7.9 | 18.5 (17.4, 19.5) | -1.9 ± 7.8 |
| **Ethnicity Specific** | | | | | | | | |
| Asian | 0.5 (0.3, 0.5) | 16.0 ± 74.6 | 4.5 (4.4, 4.5) | -3.4 ± 18.2 | 0.4 (0.4, 0.60) | 62.4 ± 95.3 | 1.4 (1.1, 1.7) | 18.1 ±52.8 |
| Black | 1.8 (1.3, 2.1) | 118.8 ± 264.1 | 6.3 (5.8, 6.6) | 0.9 ± 15.0 | 1.1 (0.8, 1.4) | *N/A | 1.7 (1.6, 1.8) | -2.3 ± 19.4 |
| Mixed and Other | 4.1 (3.6, 4.5) | 17.0 ± 44.7 | 23.2 (21.4, 25.5) | 10.5 ± 20.0 | 0.7 (0.3, 1.2) | *N/A | 4.9 (4.0, 6.1) | -2.8 ± 53.6 |
| Unknown | 15.5 (12.7, 18.6) | 4.0 ± 49.5 | 84.1 (72.1, 95.9) | -2.3 ± 34.2 | 2.5 (2.4, 4.5) | 33.4 ± 173.9 | 22.5 (19.3, 28.2) | -7.8 ± 18.2 |
| White | 4.5 (4.2, 4.6) | -0.2 ± 9.7 | 13.0 (12.4, 13.1) | -2.6 ± 7.9 | 1.0 (1.0, 1.0) | -3.8 ± 8.1 | 5.8 (5.7, 5.9) | -1.6 ± 9.2 |
| **Deprivation Specific** | | | | | | | | |
| 1 | 4.9 (4.5, 5.1) | 2.9 ± 18.0 | 18.3 (17.5, 18.9) | -1.3 ± 7.4 | 1.3 (1.3, 1.4) | 20.5 ± 49.8 | 6.3 (6.2, 6.9) | -14.1 ± 35.2 |
| 2 | 4.3 (4.2, 4.5) | 0.5 ± 10.8 | 13.8 (13.4, 14.5) | -1.7 ± 8.4 | 1.0 (0.9, 1.2) | 21.9 ± 65.1 | 5.6 (5.4, 5.8) | -13.8 ± 33.9 |
| 3 | 4.0 (3.9, 4.3) | 1.1 ± 12.9 | 12.0 (11.8, 12.6) | -0.9 ± 8.6 | 1.0 (1.0, 1.2) | 19.6 ± 39.9 | 5.5 (5.1, 5.8) | -9.7 ± 37.6 |
| 4 | 3.9 (3.8, 4.0) | 0.7 ± 6.3 | 11.1 (10.3, 11.5) | -2.8 ± 11.3 | 0.9 (0.9, 1.0) | 29.5 ± 80.1 | 5.4 (5.1, 5.6) | -12.6 ± 34.7 |
| 5 | 3.8 (3.8, 4.1) | 6.7 ± 17.2 | 9.7 (9.2, 9,7) | -1.3 ± 6.2 | 0.9 (0.9, 1.0) | 33.8 ± 57.0 | 4.9 (4.5, 5.0) | -12.3 ± 37.1 |
| **Staging Specific** | | | | | | | | |
| 1 | 0.5 (0.4, 0.5) | -1.0 ± 15.3 | 4.2 (3.7, 4.2) | 7.7 ± 25.7 | 0.2 (0.1, 0.2) | 2.7 ± 33.3 | 2.7 (2.6, 2.7) | -0.1 ± 9.3 |
| 2 | 1.0 (0.9, 1.0) | 2.4 ± 11.6 | 1.6 (1.5, 2.0) | -1.2 ± 19.8 | 0.1 (0.1, 0.2) | 4.8 ± 20.8 | 0.3 (0.3, 0.3) | 0.0 ± 13.8 |
| 3 | 1.6 (1.5, 1.7) | 9.3 ± 21.5 | 0.7 (0.6, 0.9) | 34.4 ± 68.3 | 0.2 (0.1, 0.2) | 5.7 ± 19.3 | 0.8 (0.7, 0.8) | 2.5 ± 15.3 |
| 4 | 0.4 (0.4, 0.4) | 1.2 ± 9.2 | 1.2 (1.1, 1.2) | 4.9 ± 12.9 | 0.2 (0.2, 0.2) | 2.9 ± 33.7 | 0.3 (0.3, 0.3) | 5.3 ± 14.8 |

*N/A= AAPC could not be calculated as incidence trends include values equal to 0.

**Table 3.** Table summarising average ethnicity & age specific, deprivation & age specific and age & stage specific incidences as well as average annual percentage changes (AAPC) in incidence between 2014-2020 for all anogenital cancers. Note that ethnicity —specific incidences are not available for the year 2020. The median and lower and upper quartile values are reported for the average incidences, incidences are per 100,000 people. The mean % change and standard deviation are reported for the average annual percentage change.

| | | Anal Cancer | | Cervical Cancer | | Vaginal Cancer | | Vulval Cancer | |
|---|---|---|---|---|---|---|---|---|---|
| | | Incidence | % Change | Incidence | % Change | Incidence | % Change | Incidence | % Change |
| **Ethnicity and Age Specific** | | | | | | | | | |
| Asian | <40y | 0 (0,0.1) | *N/A | 3.3 (2.8, 3.5) | -0.9 ± 39.4 | 0.1 (0, 0.2) | *N/A | 0 (0, 0) | *N/A |
| | 40-54y | 0.2 (0, 0.2) | *N/A | 4.4 (4.3, 4.6) | 4.7 ± 21.9 | 0.4 (0.2, 0.7) | *N/A | 1 (0.9, 1.5) | 79.6 ± 123.7 |
| | 55-74y | 1.2 (0.8, 1.5) | 52.6 ± 142.7 | 6.6 (6.4, 6.9) | -1.7 ± 22.9 | 0.7 (0.4, 1) | *N/A | 3 (2.0, 4.7) | 66.6 ± 110.4 |
| | 75+y | 2.3 (1.5, 3.3) | -11.6 ± 111.9 | 11.8 (9.6, 13.7) | 29.3 ± 109.8 | 2.3 (1.6, 44) | *N/A | 9.6 (6.7, 10.3) | 18.2 ± 110.6 |
| Black | <40y | 0.4 (0.1, 0.4) | *N/A | 3.6 (2.8, 4.0) | 9.7 ± 46.6 | 0 (0, 0.3) | *N/A | 0.2 (0, 0.6) | *N/A |
| | 40-54y | 2.2 (1.1, 2.4) | *N/A | 5.9 (5.7, 6.2) | 2.2 ± 8.0 | 1 (0.8, 1.2) | *N/A | 2.3 (1.8, 2.4) | -2.7 ± 37.4 |
| | 55-74y | 2.4 (1.5, 4.9) | 132.9 ± 234.5 | 7.7 (6.4, 10.3) | 14.0 ± 51.6 | 1.2 (0.2, 3.1) | *N/A | 2.7 (2.4, 4.1) | 22.4 ± 63.2 |
| | 75+y | 4.5 (0.8, 7.6) | *N/A | 20.6 (12.5, 27.6) | 230.7 ± 604.1 | 3.5 (2.7, 5.1) | *N/A | 4.7 (3.3, 5.6) | 11.1 ± 74.0 |
| Mixed and Other | <40y | 0 (0, 0) | *N/A | 17.1 (16.6, 18.1) | 0.5 ± 19.9 | 0 (0, 0) | *N/A | 0 (0, 0.5) | *N/A |
| | 40-54y | 4.7 (3.7, 5.9) | 38.0 ± 108.4 | 27.9 (20.7, 33.9) | 34.0 ± 108.4 | 0 (0, 0.9) | *N/A | 4.1 (2.9, 4.9) | 36.0 ± 148.3 |
| | 55-74y | 13.7 (10.2, 16.3) | 41.8 ± 104.4 | 36 (29.9, 46.5) | 22.4 ± 59.4 | 2.9 (0.7, 5) | *N/A | 15.9 (13.1, 19.4) | 25.0 ± 61.1 |
| | 75+y | 16.9 (10.7, 29.8) | 51.6 ± 153.5 | 31.7 (21.4, 51.9) | 73.5 ± 88.6 | 0 (0, 8.4) | *N/A | 53.1 (34.7, 62.4) | 1.8 ± 83.9 |
| Unknown | <40y | 0 (0, 1.9) | *N/A | 77.8 (61.4, 100.9) | -3.1 ± 47.7 | 0 (0.0) | *N/A | 0.5 (0, 1.2) | *N/A |
| | 40-54y | 8.5 (8.1, 9.2) | 62.4 ± 182.4 | 72.6 (67.4, 81.2) | 3.6 ± 23.6 | 0 (0, 1.5) | *N/A | 10.6 (7.1, 14) | 12.9 ± 59.1 |
| | 55-74y | 52.8 (39.6, 65.5) | 20.6 ± 54.6 | 88.1 (83.6, 107.3) | -3.7 ± 18.8 | 8.6 (4.2, 16.2) | *N/A | 64.2 (51.6, 73.3) | 9.4 ± 53.0 |
| | 75+y | 121.4 (104.5, 140.9) | 4.8 ± 48.1 | 185.7 (158, 210.1) | 18.4 ± 76.1 | 33.6 (18.3, 57.5) | *N/A | 310.2 (230.6, 343.4) | 5.7 ± 41.9 |
| White | <40y | 0.4 (0.3, 0.5) | 14.2 ± 42.8 | 20.2 (19.8, 20.4) | -7.1 ± 13.0 | 0.1 (0.1, 0.1) | -12.8 ± 17.0 | 0.6 (0.4, 0.7) | 19.0 ± 40.5 |
| | 40-54y | 3.4 (3.1, 3.6) | 4.4 ± 18.5 | 12.8 (12.4, 13.3) | 0.0 ± 10.4 | 0.6 (0.5, 0.6) | -2.4 ± 10.3 | 3.1 (2.9, 3.3) | 2.2 ± 5.1 |
| | 55-74y | 7.4 (7, 7.5) | 4.9 ± 9.7 | 8.3 (7.7, 9.0) | 0.2 ± 10.8 | 1.5 (1.3, 1.6) | 2.9 ± 22.7 | 7.2 (6.9, 7.7) | 5.1 ± 10.2 |
| | 75+y | 7.8 (7.6, 8) | -4.5 ± 20.3 | 9.1 (8.7, 9.1) | -4.3 ± 10.9 | 2.8 (2.7, 2.8) | 0.7 ± 5.7 | 18.1 (17.2, 18. | -0.4 ± 11.8 |
| **Deprivation and Age Specific** | | | | | | | | | |
| 1 | <40y | 0.5 (0.3, 0.6) | 128.8 ± 314.6 | 19.9 (19.4, 21.3) | 18.1 ± 46.9 | 0.2 (0.1, 0.3) | *N/A | 0.6 (0.3, 1) | 79.0 ± 165.1 |
| | 40-54y | 4.3 (3.6, 4.9) | 35.3 ± 78.7 | 20.9 (18.5, 21.4) | 22.4 ± 53.7 | 1 (0.5, 1.1) | 277 ± 653.9 | 4.9 (3.4, 5.3) | 6.0 ± 62.9 |
| | 55-74y | 8.4 (8, 9.2) | 27.6 ± 74.7 | 13.8 (12.8, 15.2) | 20.1 ± 42.7 | 2.2 (1.8, 2.7) | 53.5 ± 72.9 | 8.9 (6.8, 9.7) | 3.5 ± 52.4 |
| | 75+y | 10.2 (8.5, 11.2) | 28.4 ± 71.6 | 11.5 (11, 13.4) | 25.7 ± 69.1 | 4.5 (3.3, 4.6) | 45.2 ± 73.5 | 20 (14.6, 22.2) | 6.3 ± 69.9 |
| 2 | <40y | 0.3 (0.3, 0.4) | 108 ± 252.8 | 17.3 (15.6, 18.2) | 18.5 ± 54.3 | 0.1 (0.1, 0.1) | 36.1 ± 81.4 | 0.4 (0.2, 0.4) | 23.1 ± 68.7 |
| | 40-54y | 3.3 (2.8, 3.8) | 29.5 ± 66.8 | 13.5 (12.8, 14) | 21.4 ± 48.0 | 0.5 (0.5, 0.7) | 33.9 ± 90.0 | 2.6 (1.8, 3.4) | 18.0 ± 93.3 |
| | 55-74y | 8.3 (7.1, 8.3) | 25.3 ± 65.8 | 11 (10, 11.3) | 24.2 ± 51.0 | 1.3 (1.2, 1.5) | 44.3 ± 86.6 | 7.7 (5.6, 8.3) | 9.7 ± 73.8 |
| | 75+y | 8.9 (7, 10) | 29.4 ± 85.6 | 9.6 (9.4, 12.2) | 15.5 ± 48.5 | 3.2 (2.9, 3.3) | 50.5 ± 81.9 | 20.3 (13.2, 21.5) | 9.4 ± 78.7 |
| 3 | <40y | 0.4 (0.2, 0.5) | 71.1 ± 153.0 | 16.9 (12.4, 17.5) | 20.2 ± 62.3 | 0.1 (0, 0.2) | *N/A | 0.4 (0.3, 0.9) | 116.5 ± 224.4 |
| | 40-54y | 2.7 (2.3, 2.9) | 40.4 ± 106.6 | 11.8 (11.4, 12.3) | 27.7 ± 65.3 | 0.5 (0.4, 0.6) | 71.9 ± 107.8 | 3 (1.7, 3.1) | 16.4 ± 78.6 |
| | 55-74y | 7.4 (6.8, 7.7) | 32.4 ± 82.5 | 9 (8.4, 10) | 30.2 ± 68.2 | 1.6 (1.1, 2) | 39.1 ± 46.3 | 6.4 (4.4, 6.6) | 15.8 ± 87.5 |
| | 75+y | 7 (5.8, 7.5) | 18.3 ± 49.8 | 8.5 (8.1, 8.7) | 17.7 ± 42.2 | 2.9 (2.6, 3.3) | 39.0 ± 57.4 | 18.9 (12.4, 19.7) | 23.4 ± 108.3 |
| 4 | <40y | 0.4 (0.3, 0.5) | 137.1 ± 222.2 | 16 (12.8, 18.2) | 18.3 ± 72.4 | 0.1 (0, 0.2) | *N/A | 0.5 (0.2, 0.7) | 40.5 ± 55.4 |
| | 40-54y | 2.2 (2, 2.7) | 25.5 ± 60.8 | 10.1 (10, 10.6) | 19.5 ± 47.4 | 0.6 (0.4, 0.7) | 71.8 ± 139.1 | 1.8 (1.3, 2.1) | 13.9 ± 54.7 |

| | | | | | | | | | |
|---|---|---|---|---|---|---|---|---|---|
| | 55-74y | 6.1 (5.5, 6.4) | 28.4 ± 74.9 | 7.1 (6.4, 7.8) | 21.3 ± 55.0 | 1.1 (0.9, 1.2) | 41.9 ± 73.1 | 6.3 (4, 6.9) | 13.2 ± 85.7 |
| | 75+y | 6.9 (6.6, 7.5) | 24.0 ± 55.4 | 7.4 (6.8, 8.9) | 14.2 ± 44.6 | 2.6 (2.4, 3) | 55.2 ± 82.3 | 16.4 (11.8, 17.8) | 10.8 ± 79.5 |
| 5 | <40y | 0.3 (0.2, 1.2) | *N/A | 14.6 (9.3, 16.5) | 28.9 ± 74.0 | 0.2 (0.1, 0.3) | *N/A | 0.2 (0.2, 0.9) | 357 ± 911.4 |
| | 40-54y | 2.3 (1.9, 2.6) | 30.9 ± 78.1 | 8.5 (8.2, 9.9) | 27.3 ± 63.7 | 0.4 (0.3, 0.7) | 38.5 ± 40.1 | 1.7 (1.2, 2.2) | 17.3 ± 82.5 |
| | 55-74y | 6.1 (5.4, 6.5) | 39.7 ± 101.6 | 5.7 (5.2, 6.4) | 37.9 ± 98.6 | 1.3 (1.2, 1.4) | 80.2 ± 130.6 | 5.9 (3.3, 6.4) | 16.6 ± 69.8 |
| | 75+y | 7.2 (6.1, 7.8) | 44.7 ± 91.9 | 8 (6.3, 8.1) | 8.6 ± 30.5 | 2 (1.5, 3) | 83.2 ± 126.3 | 13.8 (10.4, 14.6) | -0.9 ± 54.7 |
| **Age and Stage Specific** | | | | | | | | | |
| <40y | 1 | 0.1 (0.1, 0.1) | 35.3 ± 105.3 | 7.2 (7, 7.3) | 10.7 ± 35.9 | 0 (0, 0) | *N/A | 0.4 (0.2, 0.4) | 30.5 ± 60.3 |
| | 2 | 0.1 (0, 0.1) | 34.3 ± 111.6 | 1.4 (1.1, 1.6) | -4.0 ± 25.1 | 0 (0, 0) | *N/A | 0 (0, 0) | *N/A |
| | 3 | 0.1 (0.1, 0.2) | 99.7 ± 246.7 | 0.5 (0.4, 0.6) | 33.5 ± 52.7 | 0 (0, 0) | 41.0 ± 92.1 | 0.1 (0, 0.1) | 40.4 ± 102.0 |
| | 4 | 0 (0, 0) | *N/A | 0.5 (0.5, 0.6) | 6.0 ± 16.0 | 0 (0, 0) | *N/A | 0 (0, 0) | *N/A |
| 40-54y | 1 | 0.3 (0.3, 0.4) | 9.4 ± 45.2 | 4.9 (3.9, 5) | 6.2 ± 19.6 | 0.1 (0.1, 0.1) | 6.2 ± 34.3 | 1.8 (1.7, 1.8) | 2.5 ± 15.0 |
| | 2 | 0.6 (0.6, 0.7) | -2.1 ± 23.9 | 1.6 (1.5, 2.1) | 0.1 ± 25.0 | 0.1 (0.1, 0.1) | 3.9 ± 46.9 | 0.1 (0.1, 0.2) | 10.5 ± 32.4 |
| | 3 | 1.2 (1.2, 1.4) | 13.5 ± 34.5 | 0.7 (0.6, 0.9) | 46.7 ± 90.3 | 0.1 (0.1, 0.1) | 15.0 ± 71.4 | 0.3 (0.3, 0.4) | 9.3 ± 31.8 |
| | 4 | 0.3 (0.2, 0.3) | 7.0 ± 30.3 | 1 (1, 1.2) | 9.4 ± 17.2 | 0.1 (0.1, 0.1) | 6.9 ± 32.2 | 0.1 (0.1, 0.2) | *N/A |
| 55-74y | 1 | 0.9 (0.9, 1) | -3.4 ± 18.4 | 1.8 (1.5, 2) | 8.0 ± 25.0 | 0.2 (0.2, 0.2) | 0.6 ± 29.6 | 3.5 (3.4, 3.5) | -1.0 ± 10.8 |
| | 2 | 1.6 (1.5, 1.8) | 5.7 ± 24.1 | 1.9 (1.8, 2.4) | 4.9 ± 23.3 | 0.2 (0.2, 0.2) | 5.6 ± 37.0 | 0.4 (0.4, 0.4) | -1.5 ± 23.0 |
| | 3 | 2.8 (2.7, 2.9) | 6.1 ± 18.2 | 0.8 (0.6, 1) | 30.8 ± 66.9 | 0.2 (0.2, 0.3) | 16.0 ± 60.7 | 1.1 (1, 1.1) | 5.0 ± 17.5 |
| | 4 | 0.6 (0.6, 0.7) | 0.5 ± 17.5 | 1.4 (1.4, 1.5) | 2.7 ± 10.0 | 0.3 (0.3, 0.4) | 7.9 ± 30.4 | 0.4 (0.4, 0.5) | 5.7 ± 19.6 |
| 75+y | 1 | 0.7 (0.5, 0.8) | 7.0 ± 36.8 | 0.7 (0.7, 0.8) | 3.6 ± 37.3 | 0.4 (0.3, 0.4) | 9.6 ± 34.0 | 7.9 (7.4, 8.1) | -2.2 ± 6.2 |
| | 2 | 1.9 (1.8, 2.1) | 3.2 ± 16.8 | 1.9 (1.4, 2) | -7.2 ± 27.7 | 0.3 (0.2, 0.4) | 7.0 ± 38.1 | 1.1 (1, 1.1) | -0.6 ± 12.9 |
| | 3 | 2.5 (1.9, 2.8) | 17.0 ± 43.0 | 1 (0.8, 1.2) | 39.9 ± 104.8 | 0.4 (0.3, 0.4) | -4.0 ± 15.1 | 2.5 (2.3, 2.8) | -0.7 ± 22.9 |
| | 4 | 0.8 (0.7, 0.8) | 5.7 ± 32.7 | 1.8 (1.6, 2.1) | 4.5 ± 30.4 | 0.6 (0.5, 0.6) | 1.5 ± 31.9 | 1.2 (1.1, 1.3) | 4.2 ± 28.0 |

*N/A= AAPC could not be calculated as incidence trends include values equal to 0.

**Table 4.** Fisher's Exact and Chi-square analysis for relevant categorical variable associations. Chi-square results are reported as: $X^2$ *((degrees of freedom, N = sample size) = chi-square value, p = p-value).*

| | **Anal Cancer** | | **Cervical Cancer** | | **Vaginal Cancer** | | **Vulval Cancer** | |
|---|---|---|---|---|---|---|---|---|
| | **Fisher's exact** | **$X^2$** | **Fisher's exact** | **$X^2$** | **Fisher's exact** | **$X^2$** | **Fisher's exact** | **$X^2$** |
| **Ethnicity vs Age** | **p<0.001** | - | **p<0.001** | - | **p<0.001** | - | **p<0.001** | - |
| **Ethnicity vs Staging** | p=0.15 | - | | (12, N=17854) = 35.5, **p<0.001** | p=0.10 | - | **P<0.05** | - |
| **Ethnicity vs Deprivation** | - | (16, N=5852) = 47.5, **p<0.001** | - | (16, N=17854) = 170.6, **p<0.001** | **p<0.001** | - | - | (16, N=7754) = 80.9, **p<0.001** |
| **Deprivation vs Age** | - | (12, N=5852) = 53.8, **p<0.001** | - | (12, N=17854) = 104.1, **p<0.001** | - | (12, N=1433) = 18.1, p=0.11 | - | (12, N=7754) = 147.3, **p<0.001** |
| **Deprivation vs Staging** | - | (16, N=5852) = 8.52, p=0.74 | - | (12, N=17854) = 53.2, **p<0.001** | - | (12, N=1433) = 23.5, **p<0.05** | - | (12, N=7754) = 7.67, p=0.81 |
| **Age vs Staging** | - | (9, N=5852) = 55.4, **p<0.001** | - | (9, N=17854) = 2025.4, **p<0.001** | - | (9, N=1433) = 12.3, p=0.20 | - | (9, N=7754) = 82.2, **p<0.001** |

**Table 5.** Multivariate logistic regression of patient characteristics with respect to late (stage 3 and 4) vs early (stage 1 and 2) cancer staging, age ≥55 years vs <55 years and high (quintile 1 and 2) vs low (quintile 4 and 5) deprivation.

| | | Anal Cancer | | | Cervical Cancer | | | Vaginal Cancer | | | Vulval Cancer | | |
|---|---|---|---|---|---|---|---|---|---|---|---|---|---|
| **Staging** | | **OR[1]** | **95% CI[1]** | **p-value** | **OR[1]** | **95% CI[1]** | **p-value** | **OR[1]** | **95% CI[1]** | **p-value** | **OR[1]** | **95% CI[1]** | **p-value** |
| **Age** | **<55 years** | - | - | - | - | - | - | - | - | - | - | - | - |
| | **≥55 years** | **0.82** | **0.70, 0.96** | **0.013** | **3.33** | **3.01, 3.69** | **<0.001** | 1.17 | 0.82, 1.67 | 0.4 | **1.80** | **1.50, 2.18** | **<0.001** |
| **Ethnicity** | **White** | - | - | - | - | - | - | - | - | - | - | - | - |
| | **Asian** | 1.11 | 0.46, 2.84 | 0.8 | 1.02 | 0.74, 1.39 | >0.9 | 0.68 | 0.33, 1.38 | 0.3 | 1.09 | 0.67, 1.74 | 0.7 |
| | **Black** | 0.98 | 0.56, 1.75 | >0.9 | 1.13 | 0.76, 1.65 | 0.5 | **0.34** | **0.15, 0.75** | **0.009** | 0.84 | 0.41, 1.59 | 0.6 |
| | **Mixed and Other** | 1.10 | 0.60, 2.08 | 0.8 | 1.25 | 0.92, 1.68 | 0.15 | 0.61 | 0.12, 2.78 | 0.5 | 1.35 | 0.74, 2.39 | 0.3 |
| | **Unknown** | 1.13 | 0.76, 1.68 | 0.5 | 1.13 | 0.89, 1.44 | 0.3 | 1.47 | 0.58, 4.00 | 0.4 | **1.56** | **1.09, 2.21** | **0.014** |
| **Deprivation** | **Low** | - | - | - | - | - | - | - | - | - | - | - | - |
| | **High** | 1.11 | 0.97, 1.27 | 0.12 | **1.44** | **1.29, 1.59** | **<0.001** | **1.38** | **1.02, 1.86** | **0.036** | 1.10 | 0.96, 1.25 | 0.2 |
| **Age** | | | | | | | | | | | | | |
| **Ethnicity** | **White** | - | - | - | - | - | - | - | - | - | - | - | - |
| | **Asian** | 1.14 | 0.49, 3.10 | 0.8 | **1.33** | **1.07, 1.65** | **0.009** | **0.25** | **0.13, 0.47** | **<0.001** | 0.71 | 0.47, 1.10 | 0.11 |
| | **Black** | **0.30** | **0.18, 0.50** | **<0.001** | **1.58** | **1.22, 2.05** | **<0.001** | **0.35** | **0.18, 0.69** | **0.002** | **0.20** | **0.12, 0.32** | **<0.001** |
| | **Mixed and Other** | **0.45** | **0.27, 0.77** | **0.003** | **0.72** | **0.57, 0.91** | **0.006** | 0.86 | 0.26, 3.81 | 0.8 | 0.65 | 0.39, 1.12 | 0.10 |
| | **Unknown** | **1.61** | **1.06, 2.54** | **0.031** | 0.94 | 0.81, 1.10 | 0.5 | 1.59 | 0.67, 4.68 | 0.3 | 1.14 | 0.81, 1.65 | 0.5 |
| **Deprivation** | **Low** | - | - | - | - | - | - | - | - | - | - | - | - |
| | **High** | **0.70** | **0.61, 0.80** | **<0.001** | **0.81** | **0.75, 0.87** | **<0.001** | 0.98 | 0.73, 1.32 | 0.9 | **0.56** | **0.49, 0.64** | **<0.001** |
| **Deprivation** | | | | | | | | | | | | | |
| **Ethnicity** | **White** | - | - | - | - | - | - | - | - | - | - | - | - |
| | **Asian** | 1.40 | 0.66, 3.09 | 0.4 | **1.58** | **1.26, 1.98** | **<0.001** | **5.37** | **2.40, 14.4** | **<0.001** | **1.99** | **1.35, 2.98** | **<0.001** |
| | **Black** | **3.87** | **2.09, 7.85** | **<0.001** | **5.07** | **3.51, 7.59** | **<0.001** | **3.69** | **1.76, 8.70** | **0.001** | **4.78** | **2.55, 9.98** | **<0.001** |
| | **Mixed and Other** | 1.33 | 0.79, 2.28 | 0.3 | **1.68** | **1.36, 2.10** | **<0.001** | 0.93 | 0.31, 2.72 | 0.9 | **2.47** | **1.50, 4.25** | **<0.001** |
| | **Unknown** | **0.76** | **0.55, 1.04** | **0.085** | **0.80** | **0.69, 0.92** | **0.001** | 1.08 | 0.57, 2.08 | 0.8 | 0.82 | 0.63, 1.06 | 0.13 |

[1]OR= Odds Ratio, CI= Confidence Interval
